# Public health economics: a systematic review of guidance for the economic evaluation of public health interventions and discussion of key methodological issues

**DOI:** 10.1186/1471-2458-13-1001

**Published:** 2013-10-24

**Authors:** Rhiannon Tudor Edwards, Joanna Mary Charles, Huw Lloyd-Williams

**Affiliations:** 1 Centre for Health Economics and Medicines Evaluation, Institute of Medical and Social Care Research, Bangor University, Dean Street Building, Bangor, Gwynedd LL57 1UT, UK

**Keywords:** Health economics, Public health, Published guidelines, Public sector policy, Inequalities in health, Outcome measurement

## Abstract

**Background:**

If Public Health is the science and art of how society collectively aims to improve health, and reduce inequalities in health, then Public Health Economics is the science and art of supporting decision making as to how society can use its available resources to best meet these objectives and minimise opportunity cost. A systematic review of published guidance for the economic evaluation of public health interventions within this broad public policy paradigm was conducted.

**Methods:**

Electronic databases and organisation websites were searched using a 22 year time horizon (1990–2012). References of papers were hand searched for additional papers for inclusion. Government reports or peer-reviewed published papers were included if they; referred to the methods of economic evaluation of public health interventions, identified key challenges of conducting economic evaluations of public health interventions or made recommendations for conducting economic evaluations of public health interventions. Guidance was divided into three categories UK guidance, international guidance and observations or guidance provided by individual commentators in the field of public health economics. An assessment of the theoretical frameworks underpinning the guidance was made and served as a rationale for categorising the papers.

**Results:**

We identified 5 international guidance documents, 7 UK guidance documents and 4 documents by individual commentators. The papers reviewed identify the main methodological challenges that face analysts when conducting such evaluations. There is a consensus within the guidance that wider social and environmental costs and benefits should be looked at due to the complex nature of public health. This was reflected in the theoretical underpinning as the majority of guidance was categorised as extra-welfarist.

**Conclusions:**

In this novel review we argue that health economics may have come full circle from its roots in broad public policy economics. We may find it useful to think in this broader paradigm with respect to public health economics. We offer a 12 point checklist to support government, NHS commissioners and individual health economists in their consideration of economic evaluation methodology with respect to the additional challenges of applying health economics to public health.

## Background

We live in an unequal world and know that inequalities in health and lifetime opportunity are fundamentally linked to inequalities in income. A paradox is emerging–in order to affect health gain in those groups of society that face most socioeconomic challenges, we must trade off our more familiar, if implicit, Western goal of health gain maximisation. In the UK the principle of health gain maximisation underpins the technical appraisal approach taken by the National Institute of Health and Care Excellence (NICE), [1]. NICE updated its “Guide to the methods of technology appraisal” in 2013 [2]. The updated version does not appropriate directly to public health; however, the guide maintains its position from the previous version with regards to economic evaluations. The use of cost-effectiveness, especially cost-utility analysis is preferred [2]. Cost per Quality Adjusted Life Year (QALY) estimates are favoured and appraised in accordance with the £20,000-30,000 threshold [2]. This is justified by the institute’s focus on maximising health gains [2]. In health economics, three papers have begun to shape thinking about public health economics. Kelly et al. [3] set out additional challenges of applying tools of economic evaluation to public health interventions as compared with the evaluation of clinical interventions. These challenges span multiple versus single outcomes, the effect of individual behaviour change upon the successful uptake of interventions, the difficulty in establishing cause and effect due to the multi-faceted nature of public health interventions and the high level of social variation involved in public health interventions. Weatherly et al. [4] offered additional considerations that health economists should build into their evaluations of public health interventions. These considerations being the need for other approaches due to the limited availability of randomised controlled trials, measurement of a range of outcomes beyond Quality Adjusted Life Years (QALYs), consideration of inter-sectoral costs and consequences which may include wider benefits and spill over effects, and a focus on equity. Payne et al. [5], introduced the idea of some public health interventions having the characteristics of “complex” interventions, and the subsequent need to measure a much broader range of outcomes than focus on QALYs, suggesting that capability theory may offer one way forward as a means for better capturing such wider benefits [6]. These three papers focus on, and critique, the traditional toolbox of methods of economic evaluation applied to the evaluation of Public Health interventions [7]. Looking back, in the UK, Derek Wanless challenged health economists to apply their methods of economic evaluation in a public health setting [8]. More recently, the NICE Centre for Public Health Excellence has called for health economists to think more broadly about how economics as a parent discipline in its widest sense, can help support those responsible for resource allocation decisions in Public Health. This was taken forward in a Medical Research Council (MRC) Population Health Sciences Research Network (PHSRN) funded workshop on population health economics in Glasgow, May 2012. Though consensus seems to be from the authors above, that the QALY is inadequate in a public health setting; Owen et al. [9] provide a powerful message that many public health interventions are indeed cost-effective, well below the NICE threshold of £20,000-30,000 per QALY. We observe a growing interest and expectation that public health interventions should be “cost saving” [10], hence an interest by government, local government and the NHS in return on investment analysis as an alternative to cost-effectiveness analysis [11].

However, economic evaluations of public health economics are not without challenges; therefore, there is a need of guidance in this field. To examine what guidance currently exists in the field of economic evaluations of public health economics we conducted a systematic review of UK guidance, international guidance and searched for papers offering observations from key commentators of the economic evaluation of public health interventions.

## Methods

### Literature search

We followed a PRISMA [12] approach to reporting the findings of the systematic review of published guidance for the economic evaluation of public health interventions.

PubMed, MedLine, CRD database, EconLit were searched between September and October 2012 for published guidance of economic evaluation methods for public health interventions. In addition, The Medical Research Council, Joseph Rowntree Foundation, National Institute for Health and Clinical Excellence, World Health Organisation and World Bank websites were also searched for relevant guidance and reference lists of published reviews were scrutinized (e.g. Owen et al. [9]).

Databases were searched for literature for the period 1990–2012. It was deemed appropriate to narrow the search to this time period as we wanted to include the more recent contributions and the studies found did not generally refer to articles before 1990. We restricted our search to papers published in the English language. Searches were conducted in October 2012.

The search terms used were: public health, public health economics, guidance for economic/econometric evaluation of public health interventions, challenges of public health economics, methods of public health economics, world health organisation, and health economics [see Additional file 1].

### Study selection

The following exclusion and inclusion criteria were employed during the searches.

Papers were included if:

•The paper had a reference in the title and/or abstract to the methods of economic evaluation of public health interventions.

•The paper identified key challenges of conducting economic evaluations of public health interventions.

•The paper made recommendations for conducting economic evaluations of public health interventions.

•The paper was from a national source (e.g. Government or Advisory Group policy documents and reports) or published in a peer-review journal.

Papers were excluded if;

•They were not specifically related to the economic evaluation of public health interventions.

•They did not provide guidance on the economic evaluation of public health interventions.

•They were published in a language other than English.

Papers identified by the searches were screened by reading the abstracts. Articles that matched the inclusion criteria above were obtained and read by RTE, JMC and HLW. It was also necessary to search literature by hand e.g., lists of references of guidance meeting the inclusion criteria. Information was extracted from each paper on the challenges and recommendations of methods to employ when conducting economic evaluations of public health interventions. We wanted to look as widely as possible at relevant health economics specific and public policy guidance on the evaluation of public health interventions.

### Data collection process

We developed a summary of guidance and key observations, providing the following information; author, publication date, source of published guidance (e.g., UK or International) and key points. As this review was undertaken in a public policy context with reports the more prominent type of published guidance the authors were unable to adhere to PICO guidelines, instead we provided a summary.

Results will be presented as a narrative review as the search strategy aims to identify UK and international guidance and observations from key commentators. Therefore, results are likely to contain a high level of heterogeneity, which may not permit meta-analysis.

### Assessment of theoretical underpinning

To strengthen the narrative review an assessment will be made of the theoretical underpinning of the included guidance. The theoretical paradigms will be categorised based upon whether they related to a macro/micro [13], welfarist/extra welfarist [14], capabilities [15] or behavioural economics approach [16]. These theoretical underpinnings will serve as a rationale or framework for categorising the papers. It must be noted however that no specific mention was made in the guidance as to its theoretical basis, but rather it is a judgment that we the authors have made. The first paradigm delineates between macro and micro economics. It is suggested that if a set of guidance relates to the evaluation of individual programmes then it belongs in a micro framework [13]. Otherwise if the guidance looks at outcomes on a population, whole economy level then a macro framework would apply [13]. Research on macroeconomic modelling uses information fed into general equilibrium models which connect health expenditure growth to its impact on the overall economy [17]. Health impact assessments conducted by the World Bank can be said to have a strong macro basis. Further a differentiation is made based on whether the theoretical underpinnings of the guidance relates to welfarist or extra-welfarist theory. Welfarism relates to the assumption that it is a measure of utility that’s important when measuring health, that is the utility received from the consumption of healthcare goods and services relative to other goods and services [14]. It is based on individualism and consequentialism [18]. Cost-benefit analysis is a good example of a welfarist perspective [19]. Extra-welfarism on the other hand goes beyond the study of utility and takes health itself and other non-utility measures as the unit of outcome [14]. Extra-welfarism is based on the underlying idea that rational choice and utility maximising behaviour, the underpinnings of welfarist theory are irrelevant to health behaviours [18]. Health is the maxim and not welfare. The EQ-5D [20] and concurrent measurement of QALYs provide a good example of extra-welfarist thinking). The third type of theoretical basis for guidance is the capability approach. Capabilities, according to Sen [15], are concerned with the ability to achieve functionings such as attachment, role, enjoyment, security and control. Ill health does not reduce quality of life on its own but insofar as it reduces the ability to achieve, for example, independence [21]. We assess the guidance based upon whether or not it promotes the capability approach [15] in a robust way, as an alternative to the QALY approach [5]. Finally, an underlying theory based on behavioural economics studies the behavioural aspects of economic agents and how this affects their decision making [16]. Behavioural economics does not suggest a rejection of the neoclassical approach to economics, but does advocate the psychological underpinnings of economic analysis. In this way it is argued that the assumptions that underlie theory are adapted to reflect a more realistic view of the world. Here we assess whether or not the guidance is based in behavioural economic theory.

## Results

**Figure 1:**
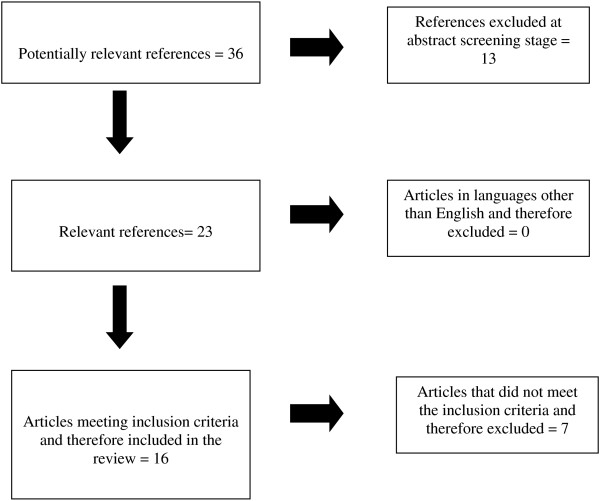
Flowchart outlining paper selection process for the systematic review.

The initial database search provided a total of 36 citations. 36 remained after removing duplicates. Of these, 13 were excluded based upon their executive summary or abstract as they did not meet the criteria. Of the remaining 23, a total of 16 papers met the inclusion criteria (See Figure 1).

Due to the heterogeneity of the results yielded by the search, we provide a narrative review below. Guidance relevant to the economic evaluation of public health interventions was divided into the following categories; UK guidance, international guidance and observations of key commentators relevant to the economic evaluation of public health interventions.

### Guidance relevant to the economic evaluation of public health interventions

#### UK guidance

Looking beyond the health economics literature to broad economic and public policy guidance, which may be directly relevant to the evaluation of public health interventions, the HM Treasury Green Book [22] outlines various points related to the treatment of costs and benefits when conducting economic appraisals of public programmes, points which relate well to the area of public health. According to the Green Book guidance, relevant costs and benefits, i.e. those which can be attributed or influenced by the intervention in question should be measured and then valued and care should be taken to avoid spuriously accurate figures [22]. Costs and benefits should be based on market prices as these are usually the best reflection of their opportunity cost i.e. best alternative use [22]. Wider social and environmental costs and benefits should be considered although there may not be a market price for these costs and benefits. However, they should not be ignored simply because it is difficult to quantify them.

When it is not possible to express costs and benefits in market prices the ‘willingness to accept measure’ should be used [22]. This is where patients state the minimum amount they would need to be compensated in order to forgo a good or service. In terms of valuing benefits the preferred measure is also market prices unless the market is distorted and does not reflect truly opportunity costs of resource use. If this is the case techniques such as ‘willingness to pay’ surveys can be used or one can infer a price from consumer behaviour through their ‘revealed preference’.

The Joseph Rowntree Foundation [23] has published a practical guide to conducting economic evaluation in the social welfare field but many of the principles can be applied to the evaluation of public health interventions. Although the study excludes health care it draws heavily upon health economics to address questions in the social welfare field. Examples of health care economic evaluation are provided but only insofar as to facilitate thinking about applying these methods in a social welfare context. There is a stronger focus on the evaluation of complex public health interventions which, according to the authors, have much more in common with the social welfare field. They outline the main methods of economic evaluation before going on to discuss methods of evaluating specific costs and benefits of different types of interventions. By providing information on how to synthesise costs and benefits they arm the reader with the necessary tools to conduct cost-effectiveness analysis.

A set of guidance has been put forward by Drummond et al. [7]. In this they acknowledge the additional challenges of applying traditional techniques of economic evaluation to a public health context over and above that of a clinical context. They add that a study by Tarn and Smith [24] concludes that there is widespread agreement on the main methods of economic evaluation of clinical interventions, whilst there is still some disagreement over specific methodological issues relating to public health. Drummond et al. [7], reiterated in Weatherly et al. [4], discussed below, identify four specific methodological challenges in economic evaluation of public health interventions:

Firstly, attributing outcomes to interventions; most published guidelines, including NICE, prefer the use of Randomised Controlled Trials (RCTs) to compare alternatives [1,2]. There are likely to be fewer controlled trials of public health programmes due to the very large sample size required to power pragmatic trials of public health interventions adequately. Follow up in clinical trials is often limited to one or two years at the most, whilst public health programmes could be expected to have an impact over the longer term. Secondly, measuring and valuing outcomes; in economic evaluations of clinical interventions, outcomes are usually measured in natural units or QALYs [1,2]. In the economic evaluation of public health interventions, other outcomes must be considered including effects on individuals not directly targeted by the programme and non-health related outcomes such as education. Some of these outcomes can be incorporated into QALYs, some not. Thirdly, incorporating equity considerations; in many cases the aim of the public health intervention is to reduce inequalities. The normal assumption in economic evaluation methods is that a QALY is of the same value to everyone who receives it. It is possible to look at the distribution of QALY gains between population sub-groups in order to tackle inequalities in health. Fourth, identifying inter-sectoral costs and consequences; the impact of public health interventions can be wide-ranging. The costs and benefits may fall on parts of the public sector not confined to health alone, such as the judicial system, education and housing.

The main guidance for the UK is set out by NICE in its 2009 document “Methods for the development of NICE public health guidance” [25]. This guidance outlines the conditions under which an economic analysis should be carried out. If a high-quality economic study has already been published which relates to the public health intervention under consideration then no further economic modelling should be attempted. However, when there is paucity of evidence in the literature and when there is expected net benefit from a public health intervention then the guidance indicates that we should proceed with economic evaluation. This guidance is underpinned by an extra-welfarist approach in that, it builds on and adapts NICE guidance for the appraisal of health care technologies [1,2], adhering to the assumption that the aim of public health interventions is to maximise QALYs. Although NICE have not explicitly recommended an ICER above which public health interventions should not be recommended and below which they should, in general, interventions with an ICER of less than £20,000 per QALY gained are considered cost-effective [25].

The Cabinet Office has published guidance on ‘Social Return on Investment’ (SROI) in public sector settings but the techniques suggested relate well to the area of public health [11]. Social Return on Investment is a method used to capture value beyond that of financial return. There are two types, evaluative and forecast. Forecast is potentially useful in the planning stage of an intervention and may show how investment could maximise impact, while evaluative SROI is retrospective. In terms of measuring costs and outcomes, this may be done using market prices. The price mechanism is an allocative concept that relates the relative valuations of a good or service by two parties. Where a cost or outcome can be measured using price there is enough information contained within that price to enable a transaction to take place where both parties maximise the benefits from the transaction. However when it is not possible to measure costs and outcomes using price, other methods have to be considered. Here the authors discuss such valuation methods, which include ‘contingent valuation’, that is the individual’s ‘willingness to pay’ (benefits) or ‘willingness to accept’ (costs) and ‘revealed preference’. After identifying and measuring the relevant costs and benefits an SROI ratio can in theory be calculated as the total present value divided by the value of inputs which gives a ratio of £x value per £1 of investment. Of course the challenge of placing monetary value on public health outcomes is not to be underestimated. It may or may not be more straightforward than in the economic evaluation of a clinical intervention, as wider impacts spilling over across sectors may in fact have market prices.

In addition to the NICE guidance referred to above there is another set of guidance published by NICE which deals with cost impact and projected return on investment of public health interventions [26]. This approach aims to measure the potential savings that can be achieved by implementing one intervention over another. The team provide tools for financial planning, over a 3–5 year time horizon to support local implementation of NICE’s recommendations. One of the main steps in the cost impact assessment is assessing interventions and identifying areas that are most likely to have a resource impact. In addition to costs of delivering services the potential savings arising from recommendations are also estimated.

In terms of the theoretical paradigms underlying UK guidance we found the majority of the guidance was classed as extra-welfarist. This guidance discussed going beyond QALYs to measure wider costs and benefits. The guidance by the Cabinet Office on SROI was guided by a micro-economic theory in that it discussed one-by-one evaluations and no attempt was made to aggregate results into a macro framework.

See Table 1 for a summary of the UK guidance above.

**Table 1:** Summary of UK published guidance

**Source of published guidance**	**Key points**	**Theoretical paradigm underpinning guidance**
HM Treasury Green Book (2003) [22]	Care should be taken to avoid spuriously accurate figures; costs and benefits should be based on market prices where possible; where not possible techniques such as ‘willingness to accept’ (for costs) and ‘willingness to pay’ (for benefits) should be used; wider social and environmental costs should be considered; use previous studies to capture value of benefits (‘benefit transfer method’).	The approach used here is a welfarist based approach whereby outcomes relate to a notion of utility.
Joseph Rowntree Foundation (Byford et al. 2003) [23]	This is a practical guide to conducting economic evaluation in the social welfare field, but many of the principles can be applied to public health interventions. There is guidance on how to measure costs and benefits along with a standard assessment of the different types of cost-effectiveness techniques (e.g., cost-effectiveness, cost-utility, cost-benefit etc.).	A welfarist approach is the theoretical underpinning here. There is no discourse as to the measuring of wider costs and benefits.
Public Health Research Consortium (Drummond et al., 2006) [7]	Acknowledges additional challenges of applying traditional techniques of economic evaluation to a public health context. Undertook a methodological review of main challenges and proposed some solutions (underpins Weatherly et al. 2009) i.e. breadth of costs and benefits; need to consider effect on inequalities in health; attribution of costs and outcomes to stakeholders; measuring and valuing outcomes across different sectors.	An extra-welfarist approach is taken here where outcomes are not confined to a welfarist utility framework, but rather a specific importance is put upon measuring wider costs and benefits which are wider than QALYs.
MRC Guidance on developing and evaluating complex interventions (2008) [32]	Defines complex interventions as “interventions with several interacting components”. Many public health interventions can be described as complex in this sense. Provides a frame work ranging from feasibility, evaluating (including assessing cost effectiveness), implementation and development. Economic evaluation is not dealt with specifically in this guidance which is focused on effectiveness.	There is no underlying theory here in terms of economics as the guidance is mainly concerned with effectiveness rather than cost effectiveness, as found in the evidence based medical movement.
NICE Public Health Guidance (2009) [25]	Economic evaluation only relevant if there is expected net benefit, taking into account number of individuals affected; uncertainty in the cost-effectiveness literature and the likelihood that economic analysis will clarify matters. Economic evaluation is not needed if: it is not possible to estimate costs and benefits; if it is obvious the resources required are modest in comparison to health gains and if robust economic evaluation is already available. Assumes that objective of public health interventions is to maximise QALY. Cost per QALY calculations recommended using a £20,000-30,000 threshold. Also cost-consequence or cost-benefit analyses recommended.	This guidance is based on a conceptual framework for public health which comprises four vectors–population, environment, society and organisations all of which affect human behaviour. An extra-welfarist approach is taken whereby QALYs are viewed from an extra-welfarist perspective.
Cabinet Office Social Return on Investment Guide (2010) [11]	There are two types of SROIs: evaluative and forecast; assigning a value to things that do not have a market price: contingent valuation–willingness to pay or accept compensation, revealed preference–hedonic pricing and average household spending–from sources such as the Family Spending Survey; SROI ratio is calculated as the total present value divided by the value of inputs–this gives a ratio of £x of value per £1 of investment; sensitivity analysis needs to be carried out–by how much would the parameters need to be changed to get an SROI of £1:£1.	SROI analysis has a microeconomic theoretical underpinning whereby evaluation of specific interventions is done on a one-by-one basis and no attempt is made to aggregate the results into a macro framework.
NICE guidance for assessing cost effectiveness and ROI (2011) [26]	A cost impact project set up by NICE in 2010. The aim is to meet public sector demands to demonstrate potential returns on investment of public health interventions. It produces tools for financial planning to support local implementation of NICE’s recommendations. This report assesses the literature in terms of cost-effectiveness analyses and their adherence to NICE guidelines. They provide a number of recommendations to changes to NICE’s methods and processes.	Wider costs and benefits are considered here rather than concentrating on QALYs alone. Insofar as this is true it could be said that an extra-welfarist theoretical approach has been taken.

Summary of UK published guidance relevant to the economic evaluation of public health interventions.

#### International guidance

We found a range of published guidance on applying economic evaluation methods to public health interventions from international organisations. For example the World Bank guidance [27] is focused on using techniques of economic evaluation to select a cost-effective package of healthcare interventions in developing countries. Total healthcare costs are expected to rise in the future due to the ageing population needing more hospital and long term care. This has implications for the financing and organisation of health services. The need for economic analysis of public health interventions has therefore never been greater. This manual provides guidelines for health planners on how to collect, analyse and interpret cost and effectiveness data to evaluate a package of health services. It cannot however take into account the inherent differences between countries in terms of different health care systems having different features and so it recommends that the approach be customised in order to accommodate country specific studies.

The World Health Organisation (WHO) [28] who advocate use of a generalised cost-effectiveness analysis approach, where the comparator is “doing nothing”, as opposed to the “usual care” allowing decision makers to see what could be achieved if all health care resources were re-allocated. So instead of advocating an “incremental” approach to evaluation, the WHO approach encourages analysis of a portfolio of interventions to see whether they provide an efficient use of resources. The WHO guidance focuses on the need to be able to generalise results across different international settings. They argue that generalised cost-effectiveness analysis can be used for a wide range of health technologies, including public health interventions.

Honeycutt et al. [29] provide guidance from the US which takes the form of a practical ‘step-by-step’ guide to conducting cost-effectiveness analyses of public health interventions. It begins by identifying the study question which is essential in order to know what costs and benefits to include and what kind of methods to employ. The guidance goes on to identify the study perspective, that is, from whose perspective is the study based? It distinguishes between a societal perspective, where all costs and benefits are considered even if no actual payment is made for some of these resources, and a narrower stakeholder perspective. The next step is to consider the time frame for the intervention. They argue that this is straightforward for initiatives that involve one-on-one contact over the course of, say, a year but more difficult if we are considering such interventions as media campaigns for example. The last step in the process is to choose the relevant method of economic evaluation. They list the different types of economic evaluation and suggest the context in which each should be applied. For example, cost analysis can be useful for choosing alternative resource mixtures that limit programme costs. Cost-effectiveness analysis answers questions about whether interventions produce outcomes that are worth the investment and should be used when comparing two or more strategies.

According to the WHO a Disability Adjusted Life Year (DALY) is a measurement of a year of ‘healthy’ life lost due to disease or premature death, or lived at a lower quality due to a disability or disease [28]. However, DALYs only reflect the presence of a medical condition which relates to certain functional limitations and do not give credit to interventions that improve the ability of individuals to live with a certain disability or disease. The WHO proposes to address this gap in the DALY approach by offering two public health relevant outcome measures 1) the activity limitation score and 2) participation limitation score, based on data from Zambia (Mont and Loeb [30]).

The Organisation for Economic Co-operation and Development (OECD) [31] has produced guidance which aims to give an economic framework to the prevention of chronic diseases. Their premise is that the prevention of chronic diseases may increase social welfare and enhance health equity. Their approach links chronic disease, caused mainly by lifestyle choices, with the performance of markets and rationality failures. Priority is given to areas of potential failure such as externalities, with the effects of individual consumption of tobacco, for example, having an effect on others (negative externality) thus justifying interventions. Information failure is another type of market failure whereby individuals lack sufficient knowledge on what constitutes an overall healthy diet for example. Rationality itself, the underpinning of well-functioning markets, can be said to have failed. They argue that this is the case when inconsistent time-preferences and lack of self-control lead to addictive behaviour thus making lifestyle changes difficult. The OECD suggests influencing choices available to individuals and seeks preventative measures where these market failures exist.

In terms of a theoretical paradigm all international guidance was classed as extra-welfarist, apart from the OECD (2008) which looked at the problem from a vantage of behavioural economics. The majority of guidance advocated cost-effectiveness analysis, whilst Mont and Loeb [30] proposed the use of DALYs rather than mortality on its own.

See Table 2 for a summary of the international guidance above.

**Table 2:** Summary of international published guidance

**Source of published guidance**	**Key points**	**Theoretical paradigm underpinning guidance**
World Bank (1993) [27]	The World Bank produced guidance focused on using techniques of economic evaluation to select a cost-effective package of healthcare interventions in developing countries.	The authors here write from the perspective of extra-welfarism, as they promote the use of cost-effectiveness analysis in their study.
World Health Organisation (2003) [28]	The World Health Organisation advocate use of a generalised cost-effectiveness analysis approach, where the comparator is ‘doing nothing’, as opposed to the more conventional ‘usual practice’.	The authors here write from the perspective of extra-welfarism as they promote the use of cost-effectiveness analysis in their study although they propose an alternative approach in terms of a generalised cost-effectiveness analysis.
Honeycutt et al. (2006) [29]	A practical ‘step-by-step’ guide to conducting cost-effectiveness analyses of public health interventions. These steps include defining the study question, identifying the study perspective, determining the time frame and analytic time period and selecting the type of economic study to conduct.	The authors focus on cost-effectiveness analysis and so it can be said that the theoretical underpinning here is extra-welfarism.
Mont and Loeb (2008) [30] Beyond DALYs: developing indicators to assess the impact of public health interventions on the lives of people with disabilities.	Aims to address a gap in the DALY approach by offering two public health relevant outcome measures 1) Activity limitation score 2) participation limitation score, based on data from Zambia.	The theoretical underpinning here is extra-welfarist since the report is concerned with improving health outcomes by using DALYs rather than mortality on its own.
OECD (2008) [31] The prevention of lifestyle related chronic diseases: an economic framework.	Focuses on the premise that prevention of chronic disease may increase social welfare and enhance health equity. This approach links chronic disease to the performance of markets and rationality failures, preventing individuals achieving the best possible outcomes. Focused on influencing choices available to individuals and seeking preventative mechanisms where market failures exist.	This approach is based on a behavioural economics theoretical framework focusing on rationality and market failure.

Summary of international published guidance relevant to the economic evaluation of public health interventions.

#### Observations of key commentators relevant to the economic evaluation of public health interventions

There have been a few publications that discuss the methodology of economic evaluations of public health interventions, most notably Kelly et al. [3], Weatherly et al. [4] and Payne et al. [5]. Kelly et al. [3] set out seven ways in which the economic evaluation of public health interventions is potentially different from the evaluation of clinical interventions. Among these is the fact that, firstly, unlike clinical interventions most public health interventions require a change in individual or population behaviour in order to ensure uptake of the intervention in the first place. This behavioural change must be modelled into the economic analysis. Another problem concerns biological and social variation, in that clinical trials are conducted in a narrowly defined area and within a known span of biological variation. Public health interventions, on the other hand, occur in a much wider socio-economic context which is not as easily defined or as generalisable. Finally, public health interventions can change during their implementation which can complicate the interpretation of results. This is because public health interventions are usually implemented without any pre-trial development and are thus susceptible to change during their implementation.

Weatherly et al. [4] suggest four key methodological challenges that face the economic analyst when conducting evaluations of public health interventions. These comprise firstly, the problem of attributing effects to a specific public health intervention and the fact that there will inevitably be less use of randomised controlled trials in the analysis of public health programmes due to power and sample size requirements. Secondly, measuring and valuing outcomes; QALYs may not be the best way to measure effects of individuals not targeted by the intervention and other non-health effects. Thirdly, equity considerations; i.e. it is possible to look at the distribution of QALY gains between population sub-groups, but that there will be a trade off or sacrifice of moving from an efficiency goal of overall QALY maximisation. Fourth, inter-sectoral costs and consequences; costs and benefits may fall on parts of the public sector not confined to health alone.

Payne et al. [5] distinguish between ‘simple’ and ‘complex’ interventions by recourse to the 2008 MRC guidance [32]. Accordingly complex interventions have the properties of involving; more than one group or organisational level that is targeted by the intervention, numerous and variable outcomes and a degree of flexibility or tailoring of the intervention being permitted. They do not talk specifically about public health interventions although these can also be deemed as complex. Rather the authors discuss complex interventions in the context of genetic services. These are regarded as similar to other types of complex interventions in that they take into account non-health outcomes as well as health related outcomes. The aim of this paper is two-fold. Payne et al. [5] seek to understand if it is reasonable to measure health status alone in the context of complex interventions. They come to the conclusion that public health guidance is more pragmatic than for clinical interventions and should include alternative outcome measures, such as life-years gained and cases averted, as well as QALYs. Therefore, maximising QALY gains should not be the only aim of complex/public interventions. It should also be looking at Sen’s capability theory [15] as a possible component of outcome measures. In so far as the capability approach (defined as the ability to function given the choice) looks to distribute capability equally across society this is fundamentally different to the idea of maximising QALYs with no regard to equity issues. Secondly, the authors aim to find out if it is possible to evaluate complex interventions using the current NICE guidelines. They conclude that outcome measures associated with a complex intervention need to be extended to include; the values of the process of delivering healthcare, non-health outcomes and capability (first proposed by Sen, [15]) to participate equally in life [33]. All these are relevant arguments to how best we approach the evaluation of public health interventions.

Marsh et al. [34] concede that economic evaluation methods developed for Health Technology Assessments (technical interventions), do not capture all the costs and benefits associated with public health interventions. They argue that the trend of using modelling techniques, and specifically a broad range of outcomes, to assess public health interventions should continue. They argue for the development of a number of ‘valuation paradigms’ such as Sen’s capability approach [15] and ‘subjective well-being’ approach [35] both of which can provide the decision maker with broader measures of value than the approaches currently on offer.

Apart from the capability approach employed in Payne et al. [5] most of the documents from key commentators are underpinned by extra-welfarism. The majority of key commentators in this field see the need and importance to go beyond a utility measure of health, and this theme is recurrent in the literature.

See Table 3 for a summary of observation by the key commentators.

**Table 3:** Summary of suggestions from key health economics commentators

**Source of published guidance**	**Key points**	**Theoretical paradigm underpinning guidance**
Kelly et al. (2005) [3]	Limited evidence of what interventions will work to reduce inequalities in health; evaluating single initiatives may fail to capture effects that rely on multiple interventions; behaviour needs to be changed in order to secure uptake of the intervention; difficult to isolate cause and effect due to multi-faceted nature of public health interventions; biological variation in clinical trials are much narrower than social variation in which public health interventions take place; public health interventions can change during their implementation; at what point is an intervention judged to have succeeded?	As they advocate the use of cost-benefit analysis it could be said that the authors come from a theoretical base of welfarism.
Weatherly et al. (2009) [4]	Attribution of effects: likely to be fewer controlled trials of public health programmes–other approaches will be necessary; measuring and valuing outcomes: other outcomes, apart from QALYs, may be relevant e.g. external outcomes not confined to the health sector alone; identifying inter-sectoral costs and consequences: costs and benefits may fall on many parts of the public sector; incorporating equity considerations: in many cases the main objective of the intervention is to reduce health inequalities.	An extra-welfarist theoretical paradigm is used here in terms of concentrating on measuring benefits not adequately captured by QALYs.
Payne et al. (2012) [5]	Valuation and Evaluation Research Theme (VERT) demonstrates that complex public health interventions have broader objectives than just health gain; maximising health gain is not a sufficient objective to achieve once cost and benefits outside the healthcare sector are recognised; public health guidance is more pragmatic than for clinical interventions and includes using alternative outcome measures (e.g. life years gained, cases averted) as well as QALYs; the authors suggest a move away from defining health benefits in terms of utility or QALY maximisation to consider non-health benefits and a measure of capability (or empowerment).	A strong theoretical basis to this guidance is the capability approach. This is coupled with an extra-welfarist perspective where wider costs and benefits are considered.
Marsh et al. (2012) [34]	Reviews methods that could be employed to capture wider range of benefits generated by public health interventions; economists need to embrace a wider set of modelling techniques to capture the effects of public health interventions the selection of which should be facilitated by the production of better data on behavioural outcomes; more valuation paradigms should be explored such as the capabilities and subjective well-being approach.	The authors take an extra-welfarist perspective whereby wider costs and benefits are considered along with a wider range of modelling techniques. Behavioural economics is also a key theoretical underpinning where modelling of behavioural outcomes is considered.

Summary of suggestions by key commentators relevant to the economic evaluation of public health interventions.

## Discussion

This systematic review yielded a total of 16 relevant papers. A paucity of material is therefore apparent in this field, especially if we compare with the economic evaluation of clinical interventions. Economic evaluations of public health interventions can be seen as a nascent academic endeavour, but the literature does identify the major methodological challenges that analysts will face when conducting such evaluations.

The main theme, reflected throughout, concerns the holistic nature of public health interventions. That is, complex public health interventions, by their very nature, deal with wider social and environmental costs and benefits than do clinical interventions and therefore there is a need to consider a much broader range of outcomes than a focus on QALYs alone [3-5,11,22,23]. This is reflected in the common theme of extra-welfarism that underpins the majority of guidance found in the review. Another salient issue identified remains on the theme of QALYs. It is apparent, from the literature [4,5], that the efficiency goal of overall QALY maximisation is not sufficient in the realms of public health interventions. The reasons for this are two-fold. First public health interventions must deal with equity considerations–a theme which is recurrent in the literature. Insofar as this is true there is a trade-off between the traditional Western notion of utility maximisation on one hand and equity issues that come to the fore on the other. Indeed Payne et al. [5] suggest Sen’s capability theory [15] as a means of including equity considerations in economic analysis of public health interventions since capability theory considers the distribution of capability across society. Secondly as the public health guidance is more pragmatic than is the case for clinical interventions then alternative outcome measures such as life-years gained and cases averted should be employed in addition to QALYs. However, although the argument that we should be looking beyond QALYs is indeed compelling it should also be made clear that QALYs, and their common currency, do provide an useful way of deeming if public health interventions are cost-effective in relation to NICE thresholds [9]–an argument that could be difficult to make without them.

### Health economics coming full circle

In the UK, health economics in the 1960s grew out of more general public policy economics, responding to a demand from the NHS to address resource allocation issues within a culture of evidence based medicine [36,37]. In the 1990s and 2000s, health economics remained within a “medical model” of healthcare [38-40], with a focus on the standardisation of methods of evaluation [7]. However, paradigms were beginning to change with a focus on “health economics” rather than health services economics [41], which has continued through the decade, with growing interest in how to apply techniques of economic evaluation to the evaluation of public health interventions. Arguably this developed from the Wanless 2004 [8] report and a growing awareness of pressures on the NHS attributable to lifestyle choices. Recent commentators (Kelly et al. [3], Weatherly et al., [4] and Payne et al., [5]) have argued for health economists to measure a full range of outcome measures, going beyond QALYs; take account of stakeholders, and to acknowledge potential impacts on the need to address inequalities in health. We argue here that health economics may have come full circle from its roots in broad public policy economics and that we may find it useful to think in this broader paradigm with respect to public health economics. Thinking within a broader paradigm also applies to the theoretical underpinnings of guidance within this field. The majority of guidance found was categorised as extra-welfarist; however, we argue that perhaps this should be widened to include more than utility such as QALYs. It should include capability theory, behavioural economics theory and in some cases return full circle to more welfarist principles.

### The QALY/SROI dilemma

There seems to be two schools of thought emerging in the literature relating to the economic evaluation of public health interventions. At this point it is worth restating that public health spans the environment, public policy, control of infectious diseases, screening, supporting behaviour change relating to consumption and lifestyle choices, and supporting government regulation in order to foster a society that promotes better health. There has been much more literature on economic evaluations of public health interventions aimed at the individual level than the potential much larger gains from environmental or legislative adaptation [42]. The extra-welfarist theoretical consensus of the guidance seems to be that QALYs are inadequate as a lone measure of outcome of public health interventions, as they fail in any way to capture the potential broad range of benefits to an individual, their family, community and society as a whole. However, the benefits of using a common accepted currency such as the QALY are illustrated by Owen et al. [9] who provide a powerful message that many public health interventions are indeed cost effective, well below the NICE threshold of £20,000-30,000 per QALY. Without the use of QALYs this argument could not be effectively put forward. Proponents of a SROI approach, which has its roots in welfarism, seem to have brushed away the 40 years that health economists have wrestled with assigning a monetary value to health outcomes. Why should this be any easier in a public health setting than in a clinical setting?

### Should we expect public health interventions to be cost saving?

The SROI approach perpetuates a growing expectation in our experience that public health interventions “need” to demonstrate that they will save money for society in the long run. This is interesting as decisions to fund surgical interventions or a new drug are not routinely subjected to this expectation of “invest to save”. Woolf et al. [10] in a US context, argue that there is a need to distinguish between measuring the outcome of a public health intervention e.g., the number of people who stop smoking in response to a particular intervention, as opposed to the wider long term social benefits of reduced prevalence of smoking in society and associated economic benefits. Woolf et al. [10] argue that there is a need to look at the cost-effectiveness of a public health prevention intervention in the same way as a clinical intervention–mindful that it may well have much wider benefits, not just expect it to be cost saving, hence levelling the playing field for resources between public health and clinical interventions.

### Design conduct and reporting issues of economic evaluations of public health interventions

The checklist below was inspired by the Drummond et al. checklist for a sound economic evaluation [43] and our own experiences of designing, undertaking and reporting economic evaluations alongside trials of public health interventions [44-47]. None of the guidance above, in our view, answers these questions systematically, to this extent our checklist is novel in distilling out key questions for consideration. The sets of guidance reviewed in this paper do provide a wide range of frameworks within which to approach the whole task of conducting economic evaluations of public health interventions. These questions, together with the sets of guidance reviewed in this paper highlight issues that need addressing in the design conduct and reporting of economic evaluations of public health interventions see Table 4. We have not tried to be prescriptive, rather to raise pertinent questions.

**Table 4:** Checklist of considerations when considering published guidance for the economic evaluation of public health interventions

1	What is the appropriate theoretical framework for analysis e.g., welfarist, extra-welfarist, capability theory?
2	What is the setting of the public health intervention under evaluation? (e.g., environmental change; infectious disease control; screening; supporting behaviour change; supporting government legislation or policy)
3	Is this best described as a primary, secondary or tertiary prevention intervention i.e. upstream or downstream?
4	What is the main agency (government; health service; local government; voluntary sector) responsible for implementation and who are the key stakeholders?
5	If this is an intervention aimed at behaviour change, what are the key levers of change (legislation; price; changing social norms; choice architecture and nudging)?
6	What is the appropriate time horizon of analysis and what is the most appropriate discount rate for costs and outcomes?
7	If the public health intervention aims to “shift the curve”, are we most interested in the centre or tails of the distribution?
8	How is this public health intervention likely to impact on inequalities in health?
9	Will subgroup analysis help identify the range of cost effectiveness estimates across different settings, delivery methods and population groups?
10	What are the main outcome measures of interest e.g. QALYs/DALYs or a large range of outcome measures relating to health and wider social outcomes?
11	How important is it to value costs, benefits and returns in monetary terms? Is it reasonable to expect the intervention to be cost saving in the short, medium or long term?
12	How relevant will it be to compare an ICER with the NICE threshold of £20,000-30,000 or an international equivalent?

### Strengths and limitations

This paper is the first to summarise the range of public health guidance available for the economic evaluation of public health interventions. We offer a checklist that highlights issues that need addressing in order to conduct economic evaluations of public health interventions and provide the most up to date summary of guidance in this field, which has the potential to be of wide benefit to health economists and other public health services researchers.

The narrative review methodology was chosen due to a high level of heterogeneity between each of the guidance documents. A potential limitation of this method is that it may have resulted in a limited review. However, we tried to minimise this by expanding our methodology and exploring the theoretical frameworks underpinning the guidance and discussing their implications.

Another potential limitation of this review is the language restriction placed in the inclusion criteria. The review identified guidance published only in English; this decision was based upon financial constraints of translation as the review was unfunded. Additionally, we did not find any guidance in languages other than English when conducting an initial scoping search of the literature.

## Conclusions

This paper is in no way prescriptive–rather it offers the first comprehensive source of published guidelines for those embarking on an economic evaluation of a public health intervention. The message from our public health colleagues is very clear–think multidisciplinary. We argue here that health economics has come, with respect to public health, full circle from its origins in wider public policy. If we approach the evaluation of public health interventions as “public policy economists”, measuring health outcomes as part of the full range of outcomes, rather than trying to stretch the “medical model” with the limitations of the health economics toolbox, we may make greater strides to inform public policy to improve health.

## Abbreviations

DALY: Disability adjusted life year; MRC: Medical research council; NICE: National institute of health and care excellence; OECD: Organisation for economic co-operation and development; PHSRN: Population health sciences research network; QALY: Quality adjusted life year; SROI: Social return on investment’; UK: United Kingdom; US: United States; WHO: World Health Organisation.

## Competing interests

The authors declare that they have no competing interests.

## Authors’ contributions

HLW conducted the initial electronic searches, articles matching the inclusion criteria were obtained and read by RTE, JMC and HLW. All authors contributed to the drafting of the manuscript and approved the final manuscript.

## Pre-publication history

The pre-publication history for this paper can be accessed here:

http://www.biomedcentral.com/1471-2458/13/1001/prepub

## Supplementary Material

Additional file 1 Search terms used for the systematic review presented in hierarchical and combination order. Click here for file
