# Supplementary material for: Public health economics: a systematic review of guidance for the economic evaluation of public health interventions and discussion of key methodological issues
Source: BMC Public Health. 2013 Oct 24;13:1001. doi: 10.1186/1471-2458-13-1001 (PMC4015185; doi:10.1186/1471-2458-13-1001)
Supplement: Additional file 1 — Search terms used for the systematic review presented in hierarchical and combination order. [file 1471-2458-13-1001-S1.doc]

Additional file 1: Search terms used for the systematic review presented in hierarchical and combination order.

01 Public health

02 Economics

03 1 and 2

04 1 and 2 and challenges

05 1 and 2 and methods

06 Guidance

07 Econ* evaluation

08 Public health interventions

09 6 and 7

10 7 and 8

11 6 and 8

12 6 and 7 and 8

13 World health organisation

14 Health economics
